# Dynamic Transcription of Long Non-Coding RNA Genes during CD4+ T Cell Development and Activation

**DOI:** 10.1371/journal.pone.0101588

**Published:** 2014-07-08

**Authors:** Fei Xia, Fulu Dong, Yi Yang, Anfei Huang, Si Chen, Di Sun, Sidong Xiong, Jinping Zhang

**Affiliations:** Institutes of Biology and Medical Sciences, Soochow University, Suzhou, Jiangsu Province, People's Republic of China; Northwestern University Feinberg School of Medicine, United States of America

## Abstract

**Background:**

Recent evidence shows that long non-coding RNA (LncRNA) play important regulatory roles in many biology process, including cell development, activation and oncogenesis. However, the roles of these LncRNAs in the development and activation of CD4^+^ T cells, which is an important component of immune response, remain unknown.

**Results:**

To predict the function of LncRNA in the development and activation of CD4^+^ T cells, first, we examined the expression profiles of LncRNAs and mRNAs in CD4^−^CD8^−^ (DN), CD4^+^CD8^+^ (DP), CD4^+^CD8^−^, and activated CD4^+^CD8^−^ T cells in a microarray analysis and verified these results by real time PCRs (qPCR). We found that the expression of hundreds of LncRNAs significantly changed in each process of developmental transition, including DN into DP, DP into CD4^+^CD8^−^, and CD4^+^CD8^−^ into activated CD4^+^ T cells. A Kendall distance analysis suggested that the expression of LncRNAs in DN, DP, CD4^+^CD8^−^ T cells and activated CD4^+^ T cells were correlated with the expression of mRNAs in these T cells. The Blat algorithm and GO analysis suggested that LncRNAs may exert important roles in the development and activation of CD4^+^ T cells. These roles included proliferation, homeostasis, maturation, activation, migration, apoptosis and calcium ion transportation.

**Conclusion:**

The present study found that the expression profiles of LncRNAs in different stages of CD4^+^ T cells are distinguishable. LncRNAs are involved in the key biological process in CD4^+^ T cell development and activation.

## Introduction

It is well-known that CD4^+^ T cells play critical roles in the adaptive immune system. These mature CD4^+^ T cells develop from common lymphoid precursors (CLP) in bone marrow. Subsequently, these cells mature into CD4^−^CD8^−^ double negative thymocytes (DN) and CD4^+^CD8^+^ double positive thymocytes (DP). These double positive cells develop into CD4^+^ and CD8^+^ single positive T cells. Once these mature CD4^+^ T cells encounter the antigen-loaded dendritic cells in periphery lymph organs or local sites, they can differentiate into Th1, Th2 or Th17 cells. Several of the cells can become regulatory T cells. Most of these polarized Th cells died. However, a minority of these activated Th cells become renewable memory CD4^+^ T cells that are able to rapidly mount a protective response upon encountering the same antigen because they are stimulated in the initiation stage [1]. A flurry of studies have been reported to explain how the CD4^+^ T cells develop from CLP to CD4^+^ single positive cells and how naïve CD4^+^ T cells can be polarized into different subsets of CD4^+^ T cells [1]–[5]. However, our understanding of the underlying molecular mechanism of CD4^+^ T cell differentiation and activation remains largely incomplete.

In addition to protein molecules, many studies already suggest that non-coding RNAs play important regulatory roles in many biological processes, including cell development, activation and oncogenesis [6]–[8]. Recently, long non-coding RNAs (LncRNAs) have been increasingly studied [9]–[12]. Genome-wide studies show that more than four thousand LncRNAs exist in mammalian species, such as mice and humans [13]–[15]. These LncRNAs function not only in normal development and homeostasis but also in some diseases [16], [17]. Because CD4^+^ T cells play pivotal roles in the immune system, we are therefore interested in studying the expression of LncRNA in CD4^+^ T cells during development and activation to provide new insights into the regulation of CD4^+^ T cells [18].

In the present study, we found that 7037, 9456, 8206 and 7847 LncRNAs were detected in DN, DP, CD4^+^CD8^−^ and activated CD4^+^ T cells, respectively. The expression of hundreds of LncRNAs changed significantly in each process of developmental transition, including DN into DP, DP into CD4^+^CD8^−^, and CD4^+^CD8^−^ into activated CD4^+^ T cells. A Kendall distance analysis suggested that the expression of LncRNAs in DN, DP, naive CD4^+^ T cells and activated CD4^+^ T cells were correlated with the expression of mRNAs in these T cells. The Blat algorithm and GO analysis suggested that LncRNAs may exert important roles in CD4^+^ T cells during development and activation such as proliferation, homeostasis, maturation, activation, migration, apoptosis and calcium ion transportation.

## Results

### Overview of the expression profiles of LncRNAs and mRNA during CD4+ T cell development and activation

**Figure 1:**
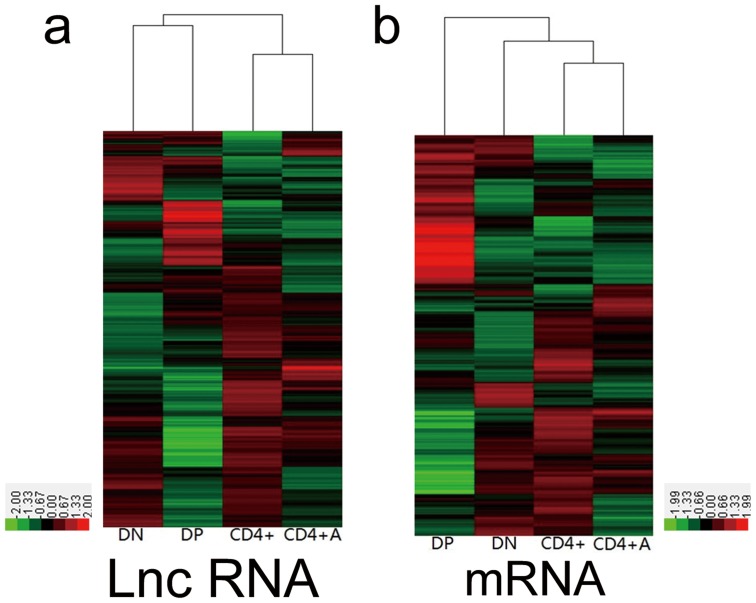
The expression profiles of LncRNAs and mRNAs in DN, DP, CD4^+^ and CD4^+^A T cells. The DN, DP, CD4^+^ and CD4^+^A T cells and their total RNA were prepared as described, the expression of (**a**) LncRNAs and (**b**) mRNAs were detected on an Agilent microarray. The raw data are displayed in a heatmap.

**Figure 2:**
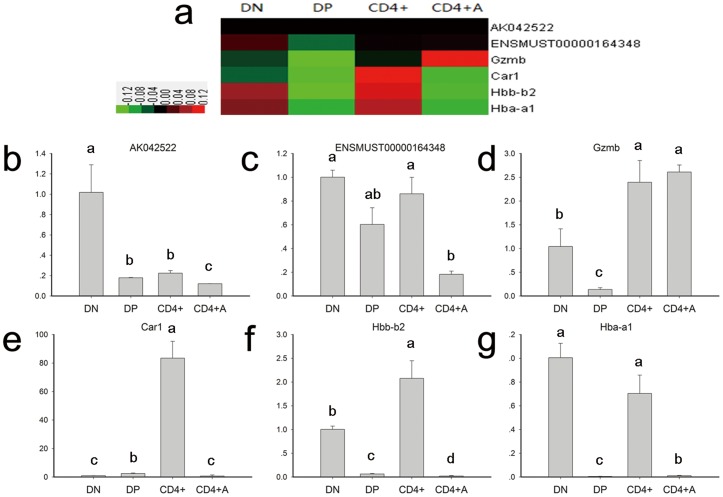
The expression profiles of two LncRNAs and four mRNAs in the T cell development and activation process. (**a**) A heatmap of two LncRNAs and four mRNAs in the T cell development and activation process. (**b–g**) The expression patterns of two LncRNAs and four mRNAs in 4 cell lines as monitored by RT-qPCR. Unlike letters associated with bars on the histogram indicate a significant difference, (P<0.05).

To explore the functions of LncRNAs in CD4^+^ T cell development and activation, we detected the expression of LncRNAs and mRNAs among the DN, DP, CD4^+^ and anti-CD3/anti-CD28 activated CD4^+^ T cells on a microarray. DN and DP cells were sorted from thymocytes, and CD4^+^ T cells were purified from the spleen. In total, 31,423 LncRNAs and 25,376 coding transcripts were detected on a second-generation LncRNA microarray (Table S1–S2). The pools of LncRNAs and mRNAs were carefully collected from databases such as RefSeq, UCSC Knowngenes, and Ensembl and from datasets published in previous studies. The organization of the expression profiles into heatmaps showed that the expression profiles of LncRNAs and mRNAs in different stages of CD4^+^ T cells were distinct (Figure 1). To examine the reliability of the array data, we randomly selected four mRNAs (Gzmb, Car1, Hbb-b2 and Hba-a1) and two LncRNAs (ENSMUST00000164348 and AK042522) to confirm their expression in different T cell subsets by using qPCR. The results of the qPCR study were consistent with the microarray data (Figure 2).

**Figure 3:**
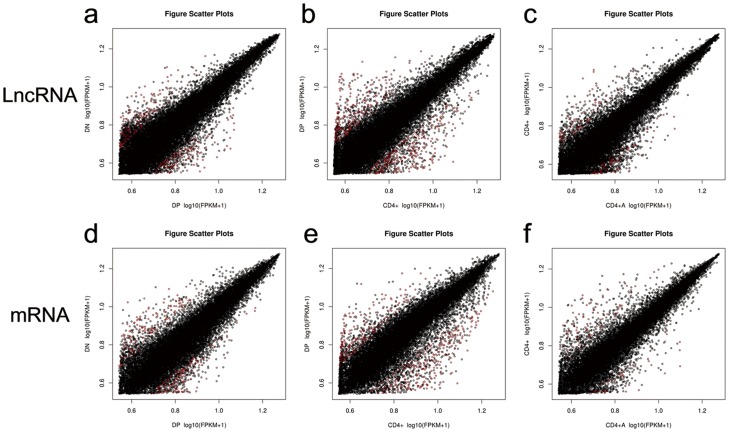
A log-log scatterplot comparing the global mRNA and LncRNA expression profiles between four subsets of CD4^+^ T cells. (**a,d**) A log-log scatter plot between DN and DP, (**b,e**) a log-log scatter plot between DP and CD4^+^, and (**c,f**) a log-log scatter plot between CD4^+^ and CD4^+^A. Red dots indicate significant differences and the black dots indicate no significant differences.

To analyze the expression changes of LncRNAs and mRNAs among DN, DP, CD4^+^ and activated CD4^+^ T cells, we grouped these cells as DN/DP, DP/CD4^+^, CD4^+^/activated CD4^+^. Additionally, we screened the LncRNA candidates and mRNAs (Table S3–S8) with the following criteria: t-test p-value ≤0.05 and fold-change≥1.5 or ≤2/3. The top 10 up- and down-regulated LncRNAs in each compare groups are shown in Table 1–3. The number of up-regulated and down-regulated LncRNAs and mRNAs in the different comparison groups are shown in Table 4. From Table 4, the number of up-regulated LncRNAs or down-regulated LncRNAs was similar to the number of mRNAs in the DN/DP and DP/CD4^+^. For example, 112 LncRNAs and 119 mRNAs were higher in expression in the DP group than those in the DN group; 131 LncRNAs and 128 mRNAs were lower in expression in the DP group than those in the DN group. We can observe this phenotype in a log-log scatter plot (Figure 3). These results may suggest that the expressions of LncRNA maintain a relationship with the expression of mRNAs in the development and activation process.

**Table 1:** The top 10 up- and down-regulated LncRNAs in the DN VS. DP group.

DN VS. DP					
UP			DOWN		
Gene name	log2(DP/DN)	p-value	Gene name	log2(DP/DN)	p-value
uc009qpv.1	1.471739597	0.03004419	uc008xuk.1	−1.417225538	0.019526053
ENSMUST00000129991	1.467481417	0.008212429	AK020242	−1.397376466	0.016699622
ENSMUST00000165285	1.457457191	0.022426522	AK007978	−1.342522112	0.006499939
uc007rul.1	1.430331255	0.002263296	AK042522	−1.291541257	0.047566101
ENSMUST00000130391	1.385793398	0.041690832	AK132374	−1.250973337	0.029195609
uc007rum.1	1.348379165	0.036369644	uc007agf.1	−1.248073538	0.033428332
ENSMUST00000156387	1.252114567	0.021579499	AK081565	−1.21586253	0.006826746
AK037315	1.234271805	0.028538955	ENSMUST00000117178	−1.196960609	0.032789358
uc009axs.1	1.227981603	0.019878953	uc009pzn.1	−1.162297221	0.030087904
ENSMUST00000130122	1.226793109	0.008816597	uc007enj.1	−1.15015982	0.028863781

**Table 2:** The top 10 up- and down-regulated LncRNAs in the DP VS. CD4+ group.

DP VS. CD4+					
UP			DOWN		
Gene name	log2(CD4+/DP)	p-value	Gene name	log2(CD4+/DP)	p-value
AK050225	1.899682846	0.020961628	ENSMUST00000144984	−1.921669086	0.00194036
AK013439	1.697413174	0.038629194	ENSMUST00000129991	−1.90189424	0.008526756
AK088994	1.623751317	0.019923999	uc008xof.1	−1.883039124	0.003175766
AK135581	1.611038693	0.001436439	uc007rum.1	−1.845952524	0.037784114
uc009sfx.1	1.580810976	0.012595544	ENSMUST00000130391	−1.815547	0.017109652
uc007dot.1	1.560820411	0.03895783	uc007kwe.1	−1.812476625	0.001288994
MM9LINCRNAEXON12112-_P1	1.546387496	0.021190725	NR_015488	−1.740748633	0.012579072
AK040987	1.534823411	0.04997947	uc009qpw.1	−1.679873533	0.019139803
AK079251	1.526350541	0.00054264	ENSMUST00000149186	−1.679190796	0.011900309
AK020242	1.505769055	0.01577034	uc007rul.1	−1.67397823	0.002808748

**Table 3:** The top 10 up- and down-regulated LncRNAs in the CD4+ VS. CD4+A group.

CD4+ VS. CD4+A					
UP			DOWN		
Gene name	log2(CD4+A/CD4+)	p-value	Gene name	log2(CD4+A/CD4+)	p-value
AK144448	1.249066165	0.047781106	ENSMUST00000100689	−1.535201274	0.040108269
AK052414	1.239167117	0.01849099	AK050225	−1.490038968	0.005443587
uc007axg.1	1.124892531	0.018913152	ENSMUST00000171480	−1.452712194	0.031353875
uc007hdw.1	1.093175797	0.002535733	MM9LINCRNAEXON12067-	−1.178812357	0.002233869
NR_033499	1.035562581	0.005349267	uc009mjt.1	−1.166556654	0.006021145
ENSMUST00000111862	1.027297815	0.012138723	AK033429	−1.163091814	0.012025539
ENSMUST00000117414	1.020519677	0.04108955	AK046352	−1.145642967	0.043572889
NR_001586	0.992395853	0.010219202	ENSMUST00000121631	−1.137694171	0.031992465
ENSMUST00000148383	0.988554886	0.010784593	uc.256+	−1.093056264	0.041327858
uc007vms.1	0.974917911	0.046199718	AK008541	−1.068485138	0.00621642

**Table 4:** The number of up- and down-regulated LncRNAs and mRNAs in the different comparison groups.

	DN VS. DP		DP VS. CD4+		CD4+ VS. CD4+A	
	LncRNA	mRNA	LncRNA	mRNA	LncRNA	mRNA
**UP**	112	119	224	228	68	36
**DOWN**	131	128	185	169	68	66
**TOTAL**	243	247	409	397	136	102

### Correlation analysis of LncRNAs and mRNAs

**Figure 4:**
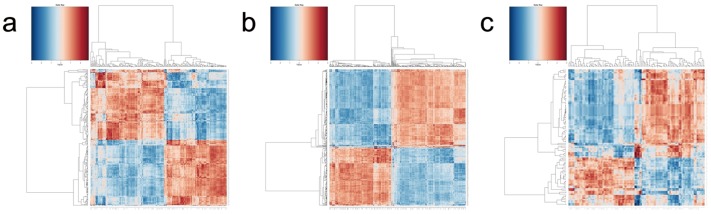
A correlation heatmap between the differentially expressed LncRNAs and genes. Rows represent differentially expressed genes, and the columns represent differentially expressed LncRNAs. A pink color indicates a positive correlation, while blue-green indicates a negative correlation. (**a**) DP VS. DN; (**b**) CD4^+^ VS. DP; and (**c**) CD4^+^A VS. CD4^+^.

To understand the relationships among selected LncRNAs and mRNAs, a Kendall distance analysis was employed to evaluate the correlation between LncRNAs and mRNAs in different comparison groups (Figure 4). In this analysis, tau is the correlation coefficient, the p-value is the significant index and a p-value≤0.05 was considered significant. By this method, we analyzed the correlation between selected LncRNAs and mRNAs in each group. We found that there were 6390, 27890, 1274 positively correlated LncRNA-mRNAs pairs in the DN/DP, DP/CD4^+^ and CD4^+^/CD4^+^A groups, respectively (p-value≤0.05), and 5699, 24824, 817 negatively correlated LncRNA-mRNAs pairs in the DN/DP, DP/CD4^+^ and CD4^+^/CD4^+^A groups, respectively (p-value≤0.05). These results suggested that the expression of LncRNAs display high correlations with mRNAs in the DP developing into single positive CD4^+^ T cell process and CD4^+^ T cells during development and activation.

### Functional prediction of LncRNAs in CD4+ T cells during development and activation

**Figure 5:**
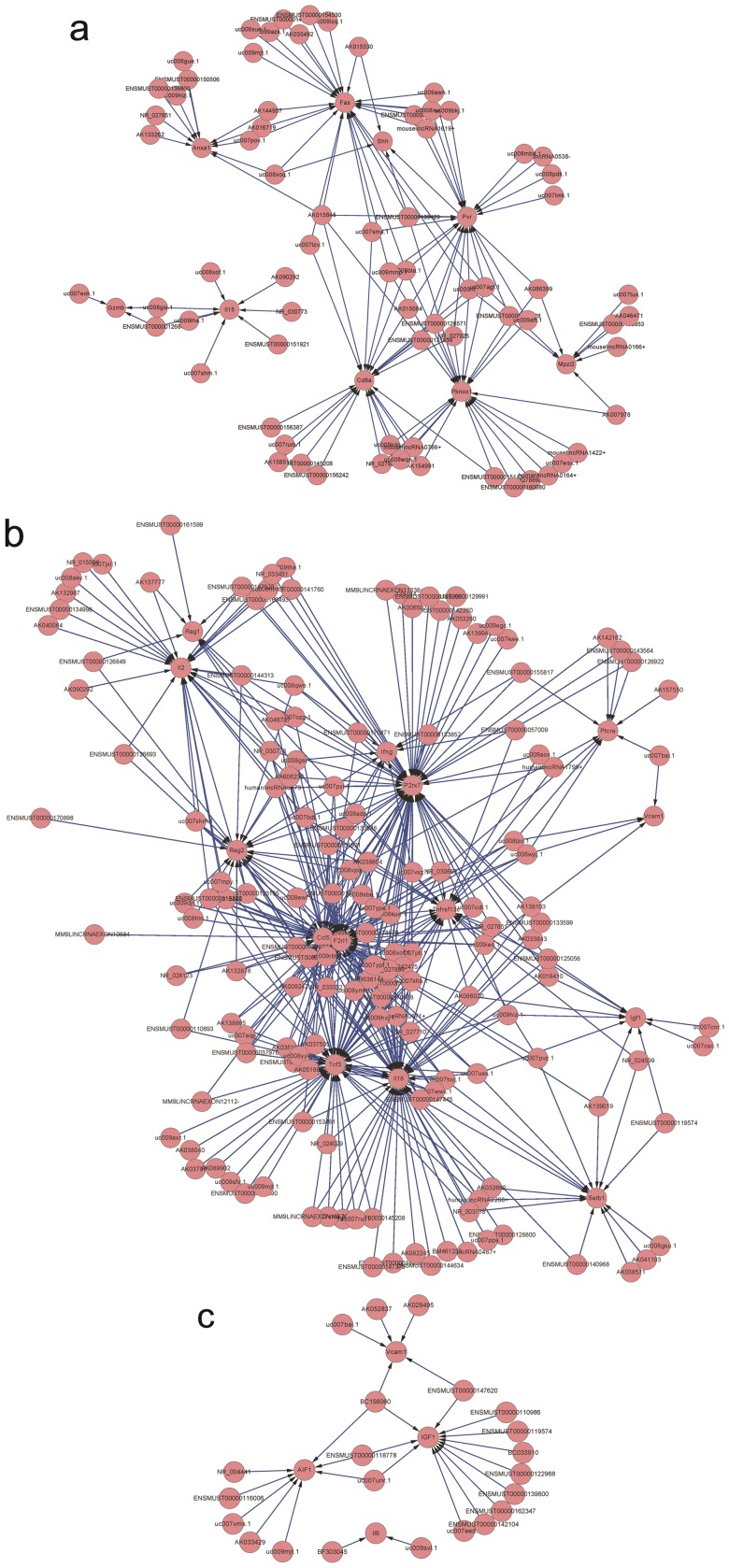
A topological graph displaying the regulation network between the LncRNAs and mRNAs. (a) DP VS. DN; (b) CD4^+^ VS. DP; and (c) CD4^+^A VS. CD4^+^.

**Figure 6:**
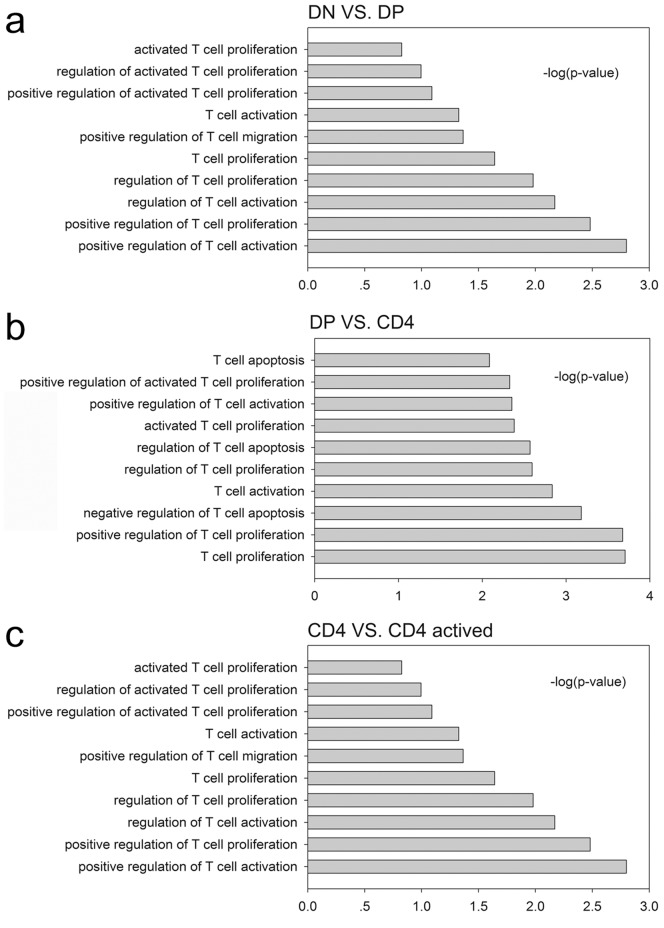
Results of the GO analysis of the genes with sequences matching LncRNAs. –log10(P) indicates the GO score related to the genes with a biological process P value. (**a**) T cell function related biological processes of the LncRNA sequence matched genes in the DP VS. DN group; (**b**) T cell function related biological processes of the LncRNA sequence matched genes in the CD4^+^ VS. DP group; (**c**) T cell function related biological processes of the LncRNA sequence matched genes in the CD4^+^A VS. CD4^+^ group.

The functional prediction of LncRNA is obscure. Numerous efforts have been pursued to predict the potential function of LncRNAs. Matching the sequence is one of the often used methods to predict the function of LncRNAs: if the sequence of a LncRNA matches a mRNA, then this LncRNA may function on this mRNA [19]. Using this hypothesis, we compared the sequences of differently expressed LncRNA 1 Kb away (upstream or downstream) from the mRNAs and the entire mRNA gene in a positively and negatively correlated group, respectively (Table S9–S26). The Blat algorithm was used for the comparison. If the LncRNA sequence matched one of the sequences 1Kb upstream or downstream of the mRNA or the mRNA gene itself (and the expression of LncRNAs and mRNAs were positively or negatively correlated), then this LncRNA potentially regulated the related mRNA. The topological graph in figure 5 demonstrates this regulation network between LncRNAs and mRNAs. For example, in the CD4^+^/CD4^+^A group, the expression of Vcam1 may be regulated by numerous LncRNAs such as uc007bai.1, AK052837, AK029495, BC156060 and ENSMUST00000147620. Meanwhile, BC156060 can regulate the expression of numerous mRNAs such as Vcam1, IGF1 and AIF1. To further predict the function of a LncRNA in CD4^+^ T cell development and activation, we performed a GO analysis with the different mRNAs that were related with LncRNAs in DN/DP, DP/CD4^+^ and CD4^+^/CD4^+^A group. In all groups, we found that LncRNA primarily regulate T cell proliferation and activation. In the DN/DP and CD4^+^/CD4^+^A groups, LncRNAs were primarily involved in the regulation of T cell migration. In the DP/CD4^+^ group, LncRNAs were primarily involved in the regulation of T cell apoptosis (Figure 6).

**Figure 7:**
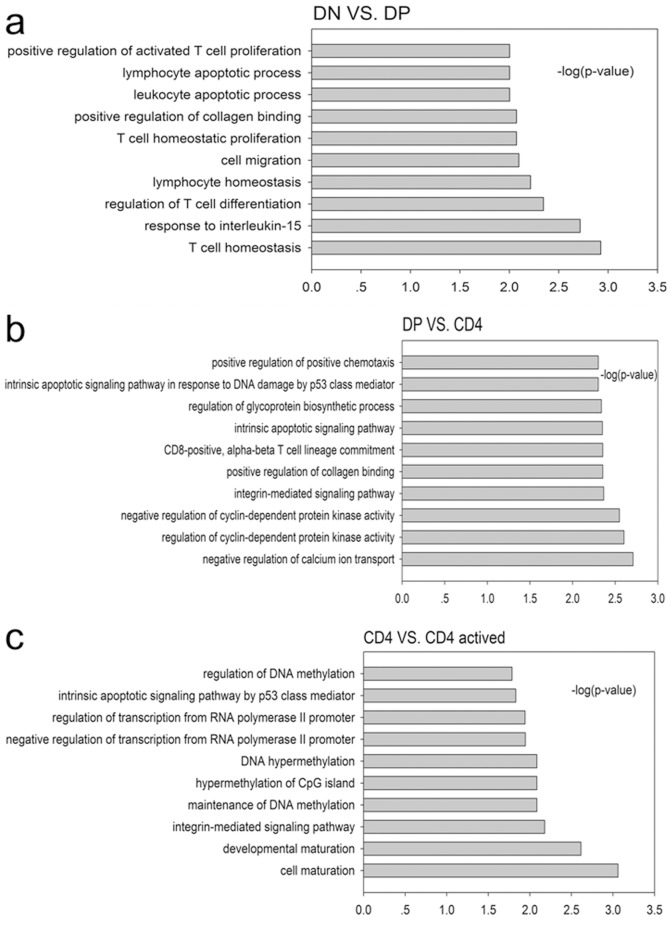
Results of the GO analysis of the genes neighboring LncRNA. –log10(P) indicates the GO score related to genes with a biological process P value. (**a**) The main biological processes of the LncRNA neighboring genes in the DP VS. DN group; (**b**) the main biological processes of the LncRNA neighboring genes in the CD4+ VS. DP group; and (**c**) the main biological processes of the LncRNA neighboring genes in the CD4^+^A VS. CD4^+^ group.

Accumulating evidence has shown that the expression of LncRNAs can regulate the expression of neighboring mRNAs, and their expressions were correlated [20], [21]. Based on this, we selected the mRNAs that neighbor LncRNAs and correlate with their expression, and we performed a GO analysis of these mRNAs in different groups. Figure 7 shows that the highest enriched biological processes were T cell homeostasis in the DN/DP group, the negative regulation of calcium ion transport in the DP/CD4^+^ group and cell maturation in the CD4/CD4^+^A group. This analysis suggested that LncRNA may be involved in these biological functions of T cells.

## Discussion

Accumulating evidence suggests that non-coding RNAs play critical regulatory roles in biological processes. Most of these studies focused on short RNAs, including small interfering RNAs (siRNAs), microRNAs (miRNAs), and PIWI-interacting RNAs. These RNAs regulate gene expression at transcriptional and/or posttranscriptional levels [22]–[24]. Recently, long non-coding RNAs have also been recognized as an important regulator in biological functions. However, our knowledge of long non-coding RNAs in immune systems is limited. Ten years ago, Deanna Haasch first showed that transcriptional activation of the BIC proto-oncogene is an early and sustained T cell activation event that suggests an important role of non-coding mRNAs in T cell function [5]. Afterward, Murad JM and colleagues demonstrated that a 200 nt ncRNA bound to PKR RNA binding site regulated gene expression in activated lymphocytes [25]. John S. Mattick's group was the first to discover long ncRNAs expressed in CD8^+^ T cells and suggested that many of these transcripts likely play roles in adaptive immunity [26]. All of these studies suggest that non-coding RNAs exert important roles in T cell activation.

In the present study, we explored the expression of long non-coding RNAs to systemically evaluate their function in CD4^+^ T cell development and activation. By using the Agilent long-coding RNA array platform, we found that the expression profiles of long non-coding RNA are significantly distinct among different stages of CD4^+^ T cells. Although expression is not necessarily indicative for function, several lines of evidence presented in this study support the likelihood that many of the LncRNAs expressed in CD4^+^ T cells are functional. First, many LncRNAs are dynamically regulated during either differentiation or activation. Second, by using a Kendall distance analysis to evaluate the correlation between the LncRNAs and mRNAs in different comparison groups (Figure 4), we found that the expression of LncRNA are highly correlated with the expression of mRNAs in CD4^+^ T cells development and activation. Notably, this high correlation was displayed in DP developing into single positive CD4^+^ T cells. Third, by using the Blat algorithm to compare the sequence match between LncRNA and mRNAs to predict the function of the LncRNA [19], we found that many LncRNAs may exert their function through certain mRNAs that play pivotal roles in T cell development and activation. For example, the sequences of several LncRNAs, including ENSMUST00000126849, ENSMUST00000161599, ENSMUST00000167528, ENSMUST00000144313, AK137777, AK046787, AK038804, and NR_030773, matched with RAG1, an important gene in T cells development. This result suggests that these LncRNAs may affect the expression of RAG1 and therefore be involved in the regulation of the arrangement of T cell receptors. The sequences of UC008xof.1, AK090292, NR_030773, ENSMUST00000151921, UC007shm.1, UC009tha.1, and UC008gsi.1 matched the sequence of IL-15 which is an important gene for T cell homeostasis, differentiation and proliferation [27]. Meanwhile, most of the evidence suggests that the expression of LncRNAs can regulate and were correlated with the expression of neighboring mRNAs [20], [21]. Based on this, we selected mRNAs, which neighbor LncRNAs and have high correlations with the expression of LncRNAs. We performed a GO analysis with these selected mRNAs for different groups. Therefore, LncRNAs may be involved in T cell maturation, homeostasis, differentiation, and apoptosis (among others) by regulating neighboring genes, including the following gene and LncRNA pairs: Runx3 and AK040461, IL-2rα and UC008iiP.1, Bax and Ak046787, Stat5b and UC0071mk.1, Myb and UC007eok.1, BCL6 and AK149396, and BCL2 and ENSMUST00000170471. However, all of these predictions only provide a starting point for the study of long non-coding RNAs in CD4^+^ T cells development and activation. The exact functions of many LncRNAs identified in this article are required experimental verification.

The present study found that the expression profiles of LncRNAs in different stages of CD4^+^ were significantly different. A bioinformatic analysis suggests that these LncRNAs may play an important role in CD4^+^ T cell development and activation. These roles may include proliferation, homeostasis, maturation, activation, migration, apoptosis and calcium ion transportation.

## Experimental Procedures

### Mice

Six- to eight-week-old C57/BL6 mice were purchased from Shanghai Laboratory Animal Company SLAC. All of these mice were maintained in the barrier facility at the Soochow University. All animal experiments were approved by the Institutional Animal Care and Use Committee of Soochow University.

### Cell preparation

Six- to eight-week-old C57/BL6 mice were sacrificed, and the thymus and spleen were harvested. The red blood cells were removed by lysis with ACK buffer. Thymus single-cell suspensions were stained with fluorescent coupled antibodies (anti-CD4 and anti-CD8) on ice for 30 min. Then, these stained samples were subjected to FACS sorting to collect the CD4^+^CD8^+^ cells as DP cells and CD4^−^CD8^−^ cells as DN cells. CD4^+^CD8^−^ T cells were purified from splenocytes using anti-CD4 microbeads according to the manufacturer's protocol (Miltenyi Biotec Inc., Auburn, California, USA). Purified CD4^+^ T cells were activated with plate-coated anti-CD3 antibodies (5 µg/mL) and anti-CD28 antibodies (2.5 µg/mL). Cells were cultured with RPMI1640 medium with 10% FCS. After 48 hr, cells were collected for further experiments.

### Antibodies

The anti-CD3 (145.11), anti-CD28 (37.51), anti-CD4-APC (GK-1.5) and anti-CD8-PE-cy7 (53–6.7) antibodies were purchased from Biolegend Inc. (San Diego, CA).

### Microarray analysis

Total RNA was extracted using TRIzol reagent (Invitrogen) according to the manufacturer's description. Total RNA from each sample was quantified using a NanoDrop ND-1000, and the RNA integrity was assessed using a standard denaturing agarose gel electrophoresis assay. For the microarray analysis, the Agilent Array platform was employed. The sample preparation and microarray hybridization were performed based on the manufacturer's standard protocols with minor modifications. Briefly, mRNA was purified from the total RNA after removing rRNA (Eukaryotic mRNA Isolation Kit, Epicentre). Each sample was amplified and transcribed into fluorescent cRNA along the entire length of the transcripts. This method avoided a 3′ bias by utilizing a reaction with random primers. The labeled cRNAs were hybridized onto the Mouse LncRNA Array v2.0 (8×60K, Arraystar). After washing the slides, the arrays were scanned on an Agilent Scanner G2505C.

Agilent Feature Extraction software (version 11.0.1.1) was used to analyze the acquired array images. Quantile normalization and subsequent data processing were performed using the GeneSpring GX v11.5.1 software package (Agilent Technologies). After the quantile normalization of the raw data, LncRNAs and mRNAs that for at least 1 out of 8 samples have flags in Present or Marginal (“All Targets Value”) were selected to remove batch effects by the Combat Software. The microarray analysis was performed by KangChen Bio-tech (Shanghai, P.R. China).

### Real time PCR

Total RNA was purified using TRIzol reagent (Invitrogen) and reverse transcribed using a reverse transcription system (Promega, Madison, WI, USA) according to the manufacturer's instructions. Real-time PCR was performed using the FastStart Universal SYBR Green Master (Roche Diagnostics, Rotkreuz, Switzerland) system and analyzed with an Eppendorf Real-Time Detection System (Eppendorf, Hauppauge, NY). The primer pairs used for Gzmb, Car1, Hbb-b2, Hba-a1, ENSMUST00000164348, AK042522 and GAPDH are shown in Table S27. The PCR parameters were as follows: 95°C for 5 min, followed by 40 cycles of 95°C for 15 sec, 60°C for 30 sec and 72°C for 30 sec. The relative expression level was calculated as 2^− (CTgapdh—CTgene)^ [28]. The mRNA level was normalized with the house keeping gene GAPDH. We used unpaired t-test to perform statistic analyses for the expression levels in different stages and p ≤0.05 were considered significant.

### Cluster analysis

Unsupervised hierarchical clustering was performed with average linkage and uncentered correlation as the similarity metrics in Cluster3.0 [29]. Heatmaps were generated in Java Treeview. The data from each raw probe on the microarrays of all four cell lines were averaged. Then, the respective data from the four cell lines were transformed as the provider divided by the average (mean). The relative expression of each gene was calculated as the ratio between the cell line microarray value and the average value of the four microarrays [30]. To draw a simple and perspicuity figure with the software, the relative expression of each gene was described as the log_10_(ratio) in the heatmap figures in Cluster3.0.

### Selection of differentially expressed mRNAs and LncRNAs

DN, DP, naïve CD4^+^ T cells and CD4^+^ activated T cells were grouped and then compared to each other to screen for the significantly differentially expressed LncRNAs and mRNAs among these four type cells. We used a paired-sample t-test and fold-change to find differentially expressed genes and LncRNAs by comparing expression levels in each group. Those mRNAs and LncRNAs with a p≤0.05 and fold-change more than 1.5 were selected.

### Analysis of the correlation between the expression of LncRNAs and mRNAs

Correlations between differently expressed LncRNAs and mRNAs in different groups were analyzed. A Kendall distance was used to calculate the correlation between LncRNA and mRNAs and the tau is the correlation coefficient. Correlations with p≤0.05 were considered significant and computed via R [31].

### Functional prediction of LncRNAs

We compared the sequence of differentially expressed LncRNAs with the sequence of mRNAs that also contained the 1 kb upstream and downstream flanking sequences. In each significant LncRNA-mRNAs correlation group, the Blat algorithm was used in the comparison. The mRNAs that matched LncRNA were further explored by a Gene Ontology (GO) analysis. We selected the LncRNA-mRNA pairs with genes functions related to T cells to draw a regulation network using Cytoscape. The –log_10_(P-value) of the GO-results were shown in the histogram. The histogram was analyzed by SigmaPlot (Systat Software, Inc., San Jose, CA, USA). We performed a GO analysis on mRNAs that were both adjacent to LncRNAs and significantly positively or negatively correlated to the expression of LncRNAs.

## Supporting Information

Table S1 **LncRNA expression profiling data.**
(XLS)

Table S2 **mRNA expression profiling data.**
(XLS)

Table S3 **DP VS. DN difference changed mRNA.**
(XLS)

Table S4 **DP VS. DN difference changed LncRNA.**
(XLS)

Table S5 **CD4+ VS. DP difference changed mRNA.**
(XLS)

Table S6 **CD4+ VS. DP difference changed LncRNA.**
(XLS)

Table S7 **CD4+A VS. CD4+ difference changed mRNA.**
(XLS)

Table S8 **CD4+A VS. CD4+ difference changed LncRNA.**
(XLS)

Table S9 **DP VS. DN LncRNA-mRNAs pairs with the expression of LncRNAs and mRNAs were positively and LncRNA sequence matched upstream of the mRNA.**
(CSV)

Table S10 **DP VS. DN LncRNA-mRNAs pairs with the expression of LncRNAs and mRNAs were positively and LncRNA sequence matched genebody of the mRNA.**
(CSV)

Table S11 **DP VS. DN LncRNA-mRNAs pairs with the expression of LncRNAs and mRNAs were positively and LncRNA sequence matched downstream of the mRNA.**
(CSV)

Table S12 **DP VS. DN LncRNA-mRNAs pairs with the expression of LncRNAs and mRNAs were negatively and LncRNA sequence matched upstream of the mRNA.**
(CSV)

Table S13 **DP VS. DN LncRNA-mRNAs pairs with the expression of LncRNAs and mRNAs were negatively and LncRNA sequence matched genebody of the mRNA.**
(CSV)

Table S14 **DP VS. DN LncRNA-mRNAs pairs with the expression of LncRNAs and mRNAs were negatively and LncRNA sequence matched downstream of the mRNA.**
(CSV)

Table S15 **CD4+ VS. DP LncRNA-mRNAs pairs with the expression of LncRNAs and mRNAs were positively and LncRNA sequence matched upstream of the mRNA.**
(CSV)

Table S16 **CD4+ VS. DP LncRNA-mRNAs pairs with the expression of LncRNAs and mRNAs were positively and LncRNA sequence matched genebody of the mRNA.**
(CSV)

Table S17 **CD4+ VS. DP LncRNA-mRNAs pairs with the expression of LncRNAs and mRNAs were positively and LncRNA sequence matched downstream of the mRNA.**
(CSV)

Table S18 **CD4+ VS. DP LncRNA-mRNAs pairs with the expression of LncRNAs and mRNAs were negatively and LncRNA sequence matched upstream of the mRNA.**
(CSV)

Table S19 **CD4+ VS. DP LncRNA-mRNAs pairs with the expression of LncRNAs and mRNAs were negatively and LncRNA sequence matched genebody of the mRNA.**
(CSV)

Table S20 **CD4+ VS. DP LncRNA-mRNAs pairs with the expression of LncRNAs and mRNAs were negatively and LncRNA sequence matched downstream of the mRNA.**
(CSV)

Table S21 **CD4+A VS. CD4 LncRNA-mRNAs pairs with the expression of LncRNAs and mRNAs were positively and LncRNA sequence matched upstream of the mRNA.**
(CSV)

Table S22 **CD4+A VS. CD4 LncRNA-mRNAs pairs with the expression of LncRNAs and mRNAs were positively and LncRNA sequence matched genebody of the mRNA.**
(CSV)

Table S23 **CD4+A VS. CD4 LncRNA-mRNAs pairs with the expression of LncRNAs and mRNAs were positively and LncRNA sequence matched downstream of the mRNA.**
(CSV)

Table S24 **CD4+A VS. CD4 LncRNA-mRNAs pairs with the expression of LncRNAs and mRNAs were negatively and LncRNA sequence matched upstream of the mRNA.**
(CSV)

Table S25 **CD4+A VS. CD4 LncRNA-mRNAs pairs with the expression of LncRNAs and mRNAs were negatively and LncRNA sequence matched genebody of the mRNA.**
(CSV)

Table S26 **CD4+A VS. CD4 LncRNA-mRNAs pairs with the expression of LncRNAs and mRNAs were negatively and LncRNA sequence matched downstream of the mRNA.**
(CSV)

Table S27 **Primers used in real time PCRs for detecting LncRNAs and mRNAs expressions.**
(DOC)
