# Supplementary material for: Dynamic Transcription of Long Non-Coding RNA Genes during CD4+ T Cell Development and Activation
Source: PLoS One. 2014 Jul 8;9(7):e101588. doi: 10.1371/journal.pone.0101588 (PMC4086894; doi:10.1371/journal.pone.0101588)
Supplement: Table S27 — Primers used in real time PCRs for detecting LncRNAs and mRNAs expressions. (DOC) [file pone.0101588.s027.doc]

**Table S27. Primers used in real time PCRs for detecting LncRNAs and mRNAs expressions**

| **accession number** | **Gene name** | **Forward primer (5'to 3')** | **Reverse primer (5'to 3')** |
| --- | --- | --- | --- |
| **NM_013542.2** | **Gzmb** | GAGAGGACTTTGTGCTGACTG | CTTAGGATTATAGTCTGGGTGG |
| **NM_008218.2** | **Hba-a1** | GACAAAAGCAACATCAAGGCTG | GCTTACATCAAAGTGAGGGAAG |
| **NM_016956.2** | **Hbb-b2** | TGGGTAATCCCAAGGTGAAGG | TGAAATCCTTGCCCAGGTGGT |
| **NM_001083957.1** | **Car1** | CACTCAGCATCTCCTATAATCC | CCACCTTTCAGAACAGATTGGT |
| **AK042522** | **AK042522** | TTCACCCTCAGGAGACCACT | CCAATGTGCCGCAGGTATGT |
| **OTTMUSG00000012445** | **ENSMUST00000164348** | TGTGAAGCGAGCAGCAAAGC | TCAACTTCATGTTCACCCCAG |
| **NM_008084.2** | **GAPDH** | AGAAACCTGCCAAGTATGATGACA | GGAAGAGTGGGAGTTGCTGTTG |
